# Risk Factors for Endothelial Decompensation after Penetrating Keratoplasty and Its Novel Therapeutic Strategies

**DOI:** 10.1155/2018/1389486

**Published:** 2018-11-15

**Authors:** Mengyuan Liu, Jing Hong

**Affiliations:** Department of Ophthalmology, Peking University Third Hospital, Beijing 100191, China

## Abstract

**Purpose:**

To review the risk factors and pathogenesis of endothelial decompensation after penetrating keratoplasty (PKP) and its novel therapeutic strategies.

**Methods:**

Literature review.

**Results:**

As the major cause of graft failure in PKP, endothelial decompensation of corneal allograft is considered an irreversible decrease in endothelial cell density and endothelial dysfunction. Various risk factors, including donor status and operative and recipient factors, have been found to be associated with this pathological process. Operative factors like graft size and recipient factors such as indications, glaucoma, or glaucoma surgery history are highly associated with the occurrence of endothelial decompensation, while others are still under investigation. Although the mechanism of these risk factors remains unclear, pathogenesis can be summarized as an acute and chronic loss of endothelium, and cell exchange between donor and recipient is at the core of chronic cell loss. Endothelial keratoplasty has been a useful alternative to repeat standard PKP in eyes with failed grafts. Descemet stripping automated endothelial keratoplasty (DSAEK) and Descemet's membrane endothelial keratoplasty (DMEK) following failed PKP provide more rapid visual recovery and achieve better rates of graft survival than those of a second PKP.

**Conclusions:**

Any direct or indirect damage to the endothelium could cause the loss, morphological changes, and dysfunction of endothelial cells. Graft size, indications, and recipient glaucoma or glaucoma surgery history are risk factors for endothelial decompensation. DSAEK and DMEK are novel therapeutic strategies for failed PKP grafts and have potential superiorities compared with repeat PKP.

## 1. Introduction

For more than half a century, penetrating keratoplasty (PKP) has been the most common allograft surgery and caters successfully to most causes of corneal blindness, including stromal or endothelial disease. Notwithstanding the rapid progression of newer keratoplasty procedures, PKP is still frequently employed worldwide [1]. The 10-year graft survival of PKP ranges from the highest, 89% for keratoconus, to the lowest, 36% for regrafts, according to the Australian Corneal Graft Registry(ACGR) (Table 1) [2–13]. Failure of the PKP graft has been a major concern of surgeons for decades, and abundant studies have shown that it is a multifactorial, progressive process, although the underlying mechanism is not fully understood. Among various causes of graft failure, endothelial failure is considered to be the major one. In addition to endothelial failure due to immune rejection in the early stage, corneal endothelial cells progressively decline over 10 years following PKP, even without rejection, which is another common cause of late failure [14, 15].

Endothelial cells lack the capacity to reproduce; any direct or indirect damage to transplanted corneal endothelial cells during or after PKP would lead to the depletion of these cells. Since the normal function of endothelial cells is crucial for the maintenance of corneal transparency, the accumulative loss of endothelial cells might finally reach a minimum threshold, followed by functional decompensation resulting in corneal edema and opacity. This irreversible reduction in endothelial cell density (ECD) and endothelial dysfunction of the corneal graft is defined as graft endothelial decompensation [16]. For decades, many scholars have investigated the etiology of the damage to corneal endothelial cells in PKP grafts, and various risk factors were found to be associated with the process of endothelial decompensation. Although the mechanism of these risk factors remains unclear, the indication for initial PKP is an important factor affecting the incidence or even the onset time of endothelial decompensation [16]. Other factors, such as intraocular pressure (IOP), graft size, and history of diabetes of the donor, have also started to draw researchers' attention, despite the controversial results of current studies [3, 7, 10, 11]. These risk factors can be classified as donor status, operative factors, and recipient factors, which have been widely adopted in numerous studies.

The patient who experiences endothelial decompensation of the PKP graft desires a second graft. Previously, all patients with endothelial failure received a repeat PKP for treatment. Unfortunately, after a repeat PKP, the patient is once again subjected to prolonged visual recovery, unpredictable refractive results, risk for suture-related complications, and an unstable full-thickness wound. Even more detrimental is that PKP has a much poorer prognosis for survival than initial keratoplasty [17]. With recent advances in lamellar corneal surgery, surgeons subsequently began to consider the option of endothelial keratoplasty (EK) for post-PKP patients with endothelial decompensation [18]. Theoretically, by only replacing the decompensated endothelium while maintaining the structural integrity of the eye, the adoption of EK to treat PKP grafts for endothelial failure seems to be an ideal procedure. In primary endothelial disease, EK has been established to be a safe therapy that offers rapid visual recovery with minimal induction of astigmatism, a mild predictable shift in refraction, less graft rejection, and fewer postoperative visits due to the suture-less nature of the procedure [19]. However, whether the advantages of EK are retained with failed PKP grafts remains uncertain. Price and Price [20] and Covert and Koenig [21] first showed that Descemet stripping automated endothelial keratoplasty (DSAEK) may be a successful technique to rapidly rehabilitate PKP grafts with endothelial decompensation. Subsequently, several case series with a longer follow-up from different centers further verified the graft survival, visual recovery, and related complications of DSAEK for the treatment of failed PKP. To date, several researchers have reported outcomes with reasonable success. Other details of the surgical procedure, such as the second graft size compared with the first one, have become the focus of current studies. Recently, Descemet's membrane endothelial keratoplasty (DMEK), a newer variation of endothelial keratoplasty, has been gradually adopted to treat failed PKP, with the superiority of achieving even faster and better visual outcomes over DSAEK [22].

In this review, we analyze the changes in the corneal endothelium after PKP and summarize risk factors for endothelial decompensation after PKP, which are classified as donor status and operative and recipient factors, as well as the pathogenesis. The novel therapeutic strategies that are used to treat failed PKP grafts, such as DSAEK and DMEK, have been validated in recent research. We also compare graft survival, visual recovery, and the occurrence of related complications, such as graft dislocation and graft rejection, among DSAEK, DMEK, and repeat PKP.

## 2. Changes in Corneal Endothelium after Penetrating Keratoplasty

Owing to the two properties of corneal endothelial cells, their function as a sodium pump to maintain transparency, and their lack of regeneration ability, the long-term optical clarity of the cornea after PKP is decided by the number, morphology, and function of donor endothelial cells [23]. However, in the presence of an irreversible ECD decline and dysfunction of the graft endothelium, endothelial decompensation of the PKP graft will occur.

### 2.1. Endothelial Cell Loss

According to previous studies, the density of endothelial cells after the first ten years following PKP has been reported to be 960 ± 470 [24], 840 ± 150 [25], or 642 ± 166 cells/mm^2^ [26], and after the next ten years, the density was 855 [27] or 666 ± 284 cells/mm^2^ [26] (Table 2 [3, 8, 9, 15, 23, 24, 26–31]). The speed of endothelial cell loss was highest in the first year after PKP, with a rate of approximately 40% [30]. Between 5 and 10 years after PKP, the rate became 4.2% per year [30]. Additionally, the corneal endothelial cells became stabilized 10 years after surgery with a rate of approximately 12% from 10 to 20 years [30]. This rate was not affected by postoperative graft rejection, causative corneal lesion, final visual acuity, and age of the recipient. Once the ECD reaches 333–500 cells/mm^2^ [29], functional decompensation will occur. Interestingly, Lass et al. recently reported that 24% of clear grafts had an ECD below 500 cells/mm^2^ in their cohort of post-PKP patients [31]. Endothelium's shifting of its glucose metabolism is a possible explanation, though the mechanism is not fully understood [31].

In recent decades, several models have been proposed to explain how ECD changes over time. The linear model assumes a constant and endothelial cell loss with time: ECD = ECD_0_ − (*t* ∗ CL), where ECD_0_ represents the preoperative ECD, *t* is the postoperative time point in years, and CL means the annual rate of endothelial cell loss [32, 33]. In the biphasic linear model, the endothelial density is described as a mixed piecewise linear model in time with a change in slope one year after surgery. The first year after surgery represents early phase endothelial cell loss, and the following years represent the late phase endothelial cell loss [32]. The first biexponential model presented by Armitage et al. suggests distinct mechanisms of endothelial decay; the fast component within the first postoperative year that persists for years is ascribed to surgical trauma, and the slow component is imputed to a chronic proapoptotic change due to alteration of the anterior chamber milieu [14, 23, 34]. However, this hypothesis cannot explain the differences in endothelial cell loss and prognosis between patients with keratoconus and bullous keratopathy [34]. Bohringer et al. proposed a new explanation for the biexponential characteristics of chronic endothelial cell loss. They supposed that the fast component reflected endothelial cells of donor origin and the slow one reflected cell loss originating from the recipient. Their hypothesis is in accordance with long-term survival in keratoconus and bullous keratopathy [34].

### 2.2. Alteration of Endothelial Morphology and Function

Endothelial cells are markedly enlarged in long-term corneal allografts, and other morphological alternations are an increased coefficient of variation (CV) of the cell area and decreased percentage of hexagonal cells (HEX) [35]. However, it remains controversial whether the changes in morphometric measures, including CV (reflecting variation in cell size) and the percentage of hexagonal cells (reflecting variation in cell shape), are predictive of subsequent endothelial failure. Benetz et al. have recently shown that, unlike ECD, changes in CV and HEX are not predictors of endothelial failure after PKP, although they are associated with cell loss [36]. Possible reasons for reducing the utility of CV and HEX can be concluded to be a compromised endothelial image quality and heterogeneous graft endothelial cells [36]. Thus, utilizing only a small sample of cells for the analysis might lead to inaccuracy and fluctuation. Other hypotheses are measurement error or an unstable status of the endothelial population in the allograft [36]. A well-designed study with a larger sample size and better image quality is needed to verify the importance of morphometric measures in predicting endothelial decompensation.

However, morphological alternations also provide a basis for endothelial dysfunction. As is commonly acknowledged, the deturgescence and clarity of the cornea depends on the pump-leak mechanism of monolayer cells lining the inner surface. The so-called pump-leak mechanism explains the two functions of the corneal endothelium, maintaining stromal deturgescence and clarity by actively pumping out fluid and forming a leaky barrier that enables fluid to leak back into the stroma. Even if their number is decreased by 80%, the function of the corneal endothelium can still be sustained [35]. Nevertheless, when endothelial cells continue to be lost while the remaining cells enlarge, pump activity is diminished. Simultaneously, resulting from the reduction of ECD, cells lining the posterior surface of the cornea decrease substantially and the intercellular space is significantly reduced, leading to a decrease in permeability. When endothelial function deteriorates and the pump-leak mechanism is destroyed, endothelial decompensation eventually ensues [29].

## 3. Risk Factors and Pathogenesis of Endothelial Decompensation

In the ACGR 2012 Report, endothelial decompensation accounted for 15% of all causes of graft failure [4]. Thus, it is important to identify the risk factors and pathogenesis to avoid endothelial decompensation after PKP. According to laboratory and clinical studies, this process is very complex. Apart from the acute immune responses leading to endothelial damage, the high rate of chronic endothelial cell loss persisting for years has become the major limitation for long-term transplant survival. Chronic endothelial cell loss for years can ultimately result in late endothelial decompensation, which is known to be the most important reason for late graft failure [37]. However, the etiology of endothelial decompensation is not fully understood and is likely a complicated interaction of several variables, including donor status, surgical techniques, and recipient factors [38]. The factors discussed below in specific are highly or possibly related factors in current studies. Other factors, such as race, cause of death, and method of retrieval, might not be associated with endothelial failure and changes in ECD, which are all summarized in Table 3 [3, 7, 10, 11, 16, 28, 31, 37–46].

### 3.1. Donor Status

#### 3.1.1. Age

Endothelial failure in corneal grafts is associated with ECD in the range from 250 to 500 cells/mm^2^, and ECD is known to decline with age [47]. Thus, it would seem intuitive that donor age and ECD should be determinants of graft survival. Nevertheless, conflicting results have been existed for years [10, 14]. In the Specular Microscopy Ancillary Study (SMAS), a slight association between greater endothelial cell loss and increasing donor age was identified [48]. However, it is worth mentioning that none of the above-mentioned cases had experienced graft failure. Lass et al. also found that younger donor age seemed to be associated with higher ECD during the first 5 years in a subset of 567 participants, especially in grafts from donors younger than forty years old [28]. However, while endothelial cell loss might be a proxy measure for later graft failure, this relationship is not straightforward [28]. SMAS researchers continuously extended the follow-up time for the next 5 years and completed the study in 2013. They found that an older donor age was indeed associated with slightly lower graft success after the first 5 years. Furthermore, a significant association was found between older donor age and greater cell loss of grafts that remained clear at 10 years [31]. Although the SMAS study has clear strengths, such as a large sample size, double-masking, and standardization of detection techniques, the observations only apply to PKP for endothelial diseases, and the end-point event is defined as graft failure rather than endothelial failure, since graft failure may not be due to endothelial failure.

Most recently, Wakefield et al. performed a large study including a total of 9415 patients after their first PKP [38]. They reported that the overall 5-year graft survival rate was 89%, free from endothelial failure. Unfortunately, there was no significant effect of donor age up to 90 years and preoperative donor ECD above the lower limit of 2200 cells/mm^2^ on endothelial failure at 5 years after PKP [38]. It appears that hazard risks are increased in the 76- to 90-year-old donor age group with a donor ECD ≤ 2600 cells/mm^2^. These data seem to be in agreement with the corneal donation policy of no upper age limit in the UK, or under 75 years of age in the US, if a lower limit of donor ECD is applied [38]. Further studies are warranted to verify the relationship between donor age, a decrease in ECD, and endothelial failure, providing adequate evidence for policy-making regarding an age limit for corneal donation.

#### 3.1.2. History of Diabetes

Many studies conducted in animals and investigating imaging, function, or cataract surgery have shown that diabetes mellitus may have harmful biochemical [49], morphological [50], and functional [51] effects on the corneal endothelium. Thus, it seems plausible to consider the donor history of diabetes as a risk factor for endothelial decompensation after PKP. However, recent accumulated clinical data from several case series seem to vary. In a subset of the Cornea Donor Study (CDS) [28] and SMAS with up to 10 years of follow-up [31], donors' diabetes history had no influence on endothelial survival following PKP. Most recently, Lass et al. examined the effect of donor historical diabetes status on graft failure and ECD ten years after PKP among 1090 enrolled subjects and found no apparent association between donor diabetes and PKP outcome [3]. The imprecise assessment of diabetes status and its complications in current studies might lead to conflicting results compared with laboratory findings; thus, a clinical trial is warranted to further clarify this conclusion. If this is the case, surgeons might change their attitude towards diabetic donors' grafts from the current reluctance to use them, which will cater to the increasing demand for keratoplasty given the growing population with diabetes.

### 3.2. Operative Factors

A larger graft size following PKP has been associated with less endothelial cell loss in bullous keratopathy and Fuchs dystrophy [45]. The study by Lass et al. also echoed this point after adjusting for baseline ECD. At 5 years, grafts larger than 8.0 to 9.0 mm in diameter experienced a median cell loss of 68% in comparison of 75% loss in grafts smaller than 7.0 to 8.0 mm in diameter and 74% for grafts 8.0 mm in diameter [28]. These authors further emphasized that this effect did not seem to be related to the disparity between the graft and recipient bed diameters [28]. Similarly, the SMAS investigated 176 participants for 10 or more years after PKP and came to the same conclusion [31]. As the donor diameter determines the ratio of the graft area to the area of the remaining host cornea, a larger graft delivers more endothelial cells and may thus provide better protection for the central graft, especially when the host endothelium is insufficient [45]. In patients with bullous keratopathy or Fuchs dystrophy in which cell migration is a prominent phenomenon, a large trephination diameter can help reduce chronic endothelial cell loss after PKP, resulting in a clear graft after cell migration from the donor to the host. However, a large graft poses a higher risk of graft rejection [28, 52]. Therefore, when using a large graft, it is important to determine the exact centration of trephination to the limbus and remove the vascularized pannus before recipient trephination to decrease the risk of immunologic reaction [45].

In addition to the graft characteristics, other possible surgical factors that influence endothelial cell loss are under discussion. In early assessments, organ culture conditions for transplant preservation were considered to cause additional endothelial cell loss. However, recent studies have illustrated the absence of statistically significant differences in postoperative rates of endothelial cell loss between corneas stored in tissue culture medium and organ-cultured corneas [37], though storage time in organ culture might affect the quality of grafts [40]. The impact of the surgeon has also been investigated. Szentmary et al. examined the short- and long-term variability of ECD after PKP among different surgeons. The results showed that individual surgical technique of the surgeon seems to impact ECD in the short-term, but no significant difference was found among surgeons in the long-term after suture removal [46].

### 3.3. Recipient Factors

#### 3.3.1. Indication

Bullous keratopathy, Fuchs dystrophy, keratoconus, and corneal scarring are considered main indications for PKP [23]. Patients with different indications experience completely different incidences of endothelial decompensation.

As relatively uncommon indications for PKP, corneal trauma and chemical and thermal burns lead to a high incidence of endothelial decompensation due to the complexity of the surgeries and lack of integrity of the eyeball. The appearance of decompensation also occurs earlier because of the serious damage and high incidence of early complications [12, 53]. Regarding congenital corneal endothelial dystrophy, patients have a relative high prevalence of rejection to damage the endothelium. On account of the young ages of these patients (3 to 7 years old), they tended to lack tolerance and presented early reactions more frequently [16]. Thus, these patients, along with patients with corneal trauma and eye burns, require an early follow-up to detect early complications and reduce the occurrence of endothelial decompensation.

For leucoma, fungal keratitis, and herpes simplex keratitis, PKP is relatively safe from endothelial decompensation [16]. For fungal keratitis, endothelial decompensation results from rejection, shallowness of the anterior chamber, and anterior synechia. Because the use of glucocorticoids for antirejection is limited in these patients, exudate and inflammatory reactions are severe. As a result, the proper use of postoperative medications for these patients merits further investigation [16]. Bullous keratopathy patients, with lower ECD on their peripheral corneas, experienced more endothelial cell loss over the 2 years after PKP, compared with keratoconus patients. The differences were not significant at 3 months but increased thereafter. The more pronounced migration of corneal endothelium in bullous keratopathy might be an explanation, which will be discussed in detail below. Since such process is gradual, this may help explain the late appearance of endothelial decompensation [45].

#### 3.3.2. Glaucoma

For decades, the relationship between glaucoma and endothelial decompensation has been a hotspot in the field of corneal transplantation. Experimental studies have demonstrated a relationship between elevated IOP and endothelial cell loss in animal models, and a clinical study in glaucoma patients with healthy corneas showed that the patients had a significantly lower ECD compared with a control group with normal IOP [54, 55]. Regarding patients undergoing PKP, glaucoma also leads to an increased loss of donor endothelial cells after transplantation following a variety of indications [37]. Due to the greater loss of endothelial cells and higher rates of rejection, graft failure rates in patients with preoperative glaucoma are two to three times greater than those without glaucoma [56]. Previous glaucoma surgery increases this risk seven times [41]. Studies also indicate that the shunt device used for glaucoma can lead to an excessive loss [57] and morphological changes [58] of corneal endothelial cells.

For IOP-related endothelial cell damage, no definite explanation has emerged to date. The pressure-induced induction of apoptosis in corneal endothelial cells may provide a possible explanation [37]. Adequately lowering of IOP is probably a promising way to sufficiently reduce graft endothelial cell loss. Regarding the shunt device-induced endothelial cell damage in glaucoma, several methods to prevent its occurrence have been explored [58, 59].

### 3.4. The Underlying Mechanism

Evidence supports that an acute loss of donor endothelium at the time of surgery results from a combination of surgical or corneal harvest trauma, and preservation damage. After keratoplasty, there is a greater rate of cell loss than the normal aging process. In all the endothelial decompensation cases that occurred more than 5 years postoperatively, 44.4% had acute rejection reaction once [16]. Because rejections are relatively common and the donor endothelium is the prime target of the specific immune-mediated attacks, the recipient's immune system recognizes the grafted tissue, then the graft's endothelial cells get destructed [60], leading to a low ECD [61]. It has been reported that once endothelial rejection occurred for once, 30%–80% of graft endothelial cells are lost, and thus only one rejection can be threatening [61].

However, even if the acute rejection can be controlled by medicine and the graft has restored transparency, loss of endothelial cells can still occur. Researchers have also observed that while 27.8% of cases show no significant clinical features indicative of rejection and the grafts remain transparent after PKP [16], after several months or years, chronic graft hypofunction that leads to graft failure can occur without any clear cause. This phenomenon has been reported in several studies and called chronic corneal allograft dysfunction (CCAD) [62, 63]. The specific mechanism of CCAD remains unclear, but previous studies have shown that it results from chronic endothelial cell loss, and endothelial dysfunction of the corneal allograft is a key process. Donor corneal endothelial cell loss is progressive for 10 years after penetrating keratoplasty. Apart from apoptosis accompanied with cell aging, cell exchange between donor and recipient is the major theory explaining the chronic endothelial cell loss [64].

Cell exchange between donor and recipient also appears to influence the rate of endothelial cell loss [37]. The human endothelium is capable of mitosis, and therefore, it is assumed that donor cells move to areas that are devoid of cells or areas of reduced cell density. Consequently, the central donor cell density must decrease over time with changes in cell size and cell shape, unless host cell replacement occurs [64]. This phenomenon provides an explanation for the higher rate of endothelial cell loss in patients with bullous keratopathy in comparison to keratoconus patients, who have a healthy endothelium with a normal cell density in the peripheral host cornea [33]. This assumption is based on host cells that can migrate across the wound to repopulate the donor or at least slow down central cell migration to the host. In vitro experiments and animal models have demonstrated centripetal and centrifugal movements of cells after keratoplasty. Using scanning electron microscopy in patient specimen, Regis-Pacheco and Binder have directly documented cell migration across the wound onto the host [64]. As the cells migrated across the wound, they tended to enlarge and spread to cover the host in areas lack of endothelium, either over bare Descemet's membrane or over fibrocellular tissue, presumably a residual of endothelial cell loss and replacement for wound healing.

However, the role of immunologic factors remains controversial, and some studies have shown that these chronic processes occur in the absence of rejection since immune effector cells have not been found [1, 16], while others interpret them as chronic subclinical immune reactions. The contribution of a chronic subclinical immune reaction to endothelial cell loss in homologous grafts has been documented [65]. By comparison of homologous and autologous grafts, Bertelmann et al. discovered a significantly lower rate of endothelial cell loss in the autologous group [65]. Likewise, Birnbaum et al. another two studies further confirmed these results [37, 66].

In summary, at the time of surgery, surgical trauma and preservation damage account for the acute loss of donor endothelium. Postoperatively, acute rejection reactions play a role in endothelial cell loss. Apart from the effect of aging, the progressive chronic cell loss for years, which finally leads to late endothelial failure, can be mainly interpreted as cell exchange between donor and recipient. The role of chronic subclinical immune reactions is also a possible mechanism. The risk factors discussed above, such as donor age, graft size, and elevated IOP [37] in recipients, all contribute to this pathological process directly or indirectly. Excessive endothelial cell loss might eventually lead to endothelial dysfunction and, ultimately, decompensation. However, further laboratory and clinical research is warranted to elucidate the pathogenesis of endothelial decompensation and clarify the exact mechanism of how these risk factors function in this pathological process.

## 4. Novel Therapeutic Strategies for Failed Penetrating Keratoplasty

As is described above, multiple factors have been associated with the endothelial failure of penetrating keratoplasty [67]. Previously, all the patients in this circumstance received repeat PKP because repeat PKP was the gold standard treatment for failed grafts. In the last few years, novel treatments are being utilized for failed PKP, including DSAEK and DMEK. However, endothelial decompensation after PKP is not a routine indication for DSAEK and DMEK, and most doctors would choose to repeat PKP on these patients instead. So, is endothelial keratoplasty a better choice for patients with endothelial decompensation following PKP? We summarize current evidence regarding this subject and comparison between the three procedures shown in recent research, although there remain some conflicts on which one should be the next step of visual rehabilitation. Further studies are needed to determine predictive factors that may help guide the decision between the three procedures.

### 4.1. Descemet Stripping Automated Endothelial Keratoplasty for Failed Penetrating Keratoplasty

By avoiding new disruptions of the corneal architecture and previous surgical scars, DSAEK represents a less-invasive procedure in comparison to repeat PKP [68]. Previous studies have demonstrated advantages of DSAEK for the treatment of primary endothelial diseases such as Fuchs dystrophy and bullous keratopathy. Concerning failed PKP grafts, the treatment effect of DSAEK has also been investigated in recent studies, including graft survival, visual recovery, and the occurrence of related complications.

#### 4.1.1. Graft Survival and Its Associated Factors

Covert and Koenig reported a prospective surgical case series of 7 eyes of 7 consecutive patients undergoing DSAEK for graft failure after PKP [21]. Except for one eye that suffered recurrent graft dislocation, all 6 remaining grafts remained clear with an average follow-up of more than 1 year [21]. Subsequently, in 2011, Anshu et al. performed a retrospective study of 60 cases and reported graft survival rates of 98%, 90%, 81%, and 74% at 1, 2, 3, and 4 years, respectively [68], exceeding previously reported survival rates for PKP regrafts [12, 69]. In 2014, Mitry et al. performed a multicenter retrospective case series with 246 patients recruited from 6 centers [70]. A total of 246 consecutive eyes that underwent DSAEK after failed PKP, with a minimum follow-up period of 1 month, were included. After a median of 17 months, 19.1% of the grafts had failed. The cumulative probability of DSAEK survival after a failed PKP graft was 0.89, 0.74, and 0.47 at 1 year, 3 years and 5 years, respectively [70]. These results indicate that DSAEK following failed PKP achieves rates of graft survival that are better or at least comparable to those of a second PKP.

Risk factors for graft failure in DSAEK after failed PKP have also started to become a focus of many researchers. Straiko et al. recommended that an oversized DSAEK graft could negatively affect its long-term survival [18]. However, Price et al. disagreed with this claim and argued that large (8.5 to 9.0 mm) primary DSAEK grafts had an excellent 5-year survival [71]. As the posterior corneal diameter is greater than the anterior corneal diameter, they supposed that it could easily support a larger diameter DSAEK than PKP and larger diameter grafts carrying larger healthy donor endothelium. Unfortunately, the multicenter study by Mitry et al. showed that the endothelial keratoplasty donor diameter was not a predictor of graft failure [70]. Further assessments are needed to determine the effect of DSAEK diameter on long-term survival for various indications. Previous studies evaluating PKP graft failure have shown that graft survival is reduced significantly in eyes with glaucoma shunt devices [72, 73]. The study by Anshu et al. in eyes undergoing DSAEK following a failed PKP echo this point that prior glaucoma tube shunt surgery is a significant risk factor [68]. Rejection before PKP failure is also a significant risk factor for subsequent DSAEK failure [70]. Other factors, such as the number of multiple prior grafts, lens status and treatment with oral corticosteroids before DSAEK, ocular surface disease, postoperative development of a persistent epithelial defect or microbial keratitis, and corneal neovascularization, are not significant [68, 70].

#### 4.1.2. Visual Recovery

In 2006, Price and Price first reported that six out of seven patients undergoing endothelial keratoplasty after PKP experienced an improvement in visual acuity (VA) [20]. Another case series of seven patients with DSAEK after PKP reported by Covert et al. noted that the postoperative VA of each case improved, and 2/3 of the patients had a best-corrected visual acuity (BCVA) of at least 20/40. In addition, BCVA was achieved rapidly within 3 months of surgery, and no patient experienced additional anisometropia or wound healing problems [21]. Likewise, Jangi et al. [74] and Lee et al. [67] observed a rapid visual rehabilitation at 3 and 6 months postoperatively. The advantage of rapid visual rehabilitation is extremely encouraging to eyes that have undergone prior PKP as they have recently experienced a prolonged visual recovery period and are anticipated to exhibit more rapid visual recovery. A larger series of 60 cases by Anshu et al. provided more exciting results [68]. The authors concluded that the visual results of DSAEK under failed PKP were equal to or even better than previously reported results of repeat PKP. However, the primary interest of the patient is the likelihood of attaining best-ever documented visual acuity (BDVA) of their previously clear grafts. In 2015, a cohort study provided an answer to this concern by demonstrating that DSAEK not only improves visual acuity in patients with failed PKP but, most importantly, allows these patients to regain their BDVA with their previous PKP [75].

#### 4.1.3. Complications

The most frequent endothelial keratoplasty complication is early graft detachment either performed as a primary procedure or regraft [76]. Therefore, identification of the root causes and best methods to prevent detachment is of particular interest. The dislocation rates in EK after PKP are reported to range from 5.3% to 43%, respectively [18, 20, 21, 68, 77]. Jangi et al. noted that the dislocation rate in cases undergoing DSAEK following failed PKP was only slightly higher than the overall dislocation rate in their personal series of all DSAEKs [74].

Factors contributing to the graft detachment have been discussed in numerous studies. The optimal sizing of the donor DSAEK grafts has been conflicting. Straiko et al. analyzed 17 eyes undergoing DSAEK after PKP and concluded that the use of smaller and equal DSAEK grafts could allow improved vision with a low rate of graft detachment [18]. However, an editorial written by Price et al. disagreed with this idea, reporting that in 57 cases using a DSAEK graft with a diameter approximately 1 mm larger than the failed PKP graft, the detachment rate was only 5.3%, which was similar to that observed by Straiko with smaller grafts [71]. Other studies have also noted that larger DSAEK grafts can fold and conform well even to the very irregular posterior surface of the PKP wound [68, 77]. Price et al. have theorized that size may not be the root cause of DSAEK detachment in failed PKP when researchers achieve similar detachment rates using grafts with completely different sizes [71]. Recently, some researchers have noted that the root cause of detachment might be either a complete initial lack of air fill to press the graft firmly into place or hypotony in the early postoperative period. Jangi et al. agreed with this point and demonstrated that eyes that had less “intact” anterior segments, such as those that had undergone prior complicated cataract extraction or previous glaucoma surgery, tended to have more iris abnormalities [74]. These eyes tended to preserve air less adequately than their counterparts, resulting in an increased risk of graft dislocation. The findings of Straiko et al. were also in accordance with the above hypothesis. They reported that in one eye experienced a wound leak that caused early postoperative hypotony, detachment of the graft occurred [18]. Price et al. have indicated that every time when the hypotony eye blinks, the eyelid will indent the corneal surface, make the graft move away from the recipient cornea, and thus lead to detachment when fluid is allowed into the interface [71]. However, future studies are needed to verify this hypothesis. Additionally, there is no clear consensus regarding the necessity of stripping Descemet's membrane to avoid DSAEK detachment [18, 20, 68, 77].

Other than graft dislocation, few detail additional complications, such as graft rejection, elevated postoperative IOP, infection, or epithelial ingrowth, have been reported. Jangi et al. reported a graft rejection rate of 3.5%, with no cases of infection or epithelial downgrowth [74]. However, in their two previous studies of primary DSAEK cases, rejection rates of 0.8% [78] and 8.5% [79] were reported. Jordan et al. [80] and Price et al. [81] performed Kaplan–Meier survival analysis and noted that the rejection rate was 3.6% after 6 months, 7.6% within 1 year, and 12% within 2 years. Thus, the rate of graft rejection did not seem to be significantly increased in patients undergoing DSAEK after PKP. Jangi et al. also reported that 26.7% of patients undergoing post-PKP DSAEK had elevated IOPs requiring additional glaucoma therapy postoperatively [74]. Banitt and Chopra noted that the incidence of glaucoma after primary DSAEK varied from 0% to 18% [82], and Vajaranant et al. reported elevated IOPs in 35% of patients with no history of glaucoma and in 45% of those with a preexisting diagnosis of glaucoma [83]. Despite the relatively small size of current studies, DSAEK after PKP does not seem to provide an increased incidence of elevated IOP compared with DSAEK in patients without previous PKP.

### 4.2. Descemet's Membrane Endothelial Keratoplasty for Failed Penetrating Keratoplasty

Recently, Descemet's membrane endothelial keratoplasty (DMEK) has revolutionized the treatment of endothelial dysfunction, and thanks to excellent clinical results, indications for DMEK have been extended [84]. So far, four previous studies have referred to the outcome and technique of DMEK following PKP. Despite the relatively small cohorts and heterogeneity in study populations, all the studies on DMEK for failed PKP grafts highlight the success in restoring corneal endothelial cell function.

In 2013, Anshu et al. first reviewed 4 patients with failed PKP who were managed with DMEK surgery and reported that the patients showed improvements in vision with a median BCVA of 20/50 as early as 1 month postoperatively [85]. Gundlach et al. analyzed the clinical data of 5 patients and reported that all patients had increased visual acuity [84]. More recently, by observing 19 eyes, Heinzelmann et al. reported that visual acuity increased from 0.05 to 0.1 in 16 eyes and central corneal thickness significantly decreased, without any major complications such as endophthalmitis or expulsive bleeding [86]. Thus, DMEK may be viable options for these patients, providing rapid visual recovery.

With avoidance of full-thickness incisions and multiple sutures on the cornea, DMEK offers great advantages over repeat PKP similar to those of DSAEK. Gundlach et al. also observed more benefits with DMEK than repeat PKP, including fewer postoperative complications associated with the suture, stable refraction, a lower rejection rate, and a shorter duration of local steroid therapy [84]. Heinzelmann et al. reported a rate of immune reactions of 11% within two years following DMEK for failed grafts, which seemed to be lower than that after repeat PKP or even DSAEK for failed PKP grafts [86]. However, the most notable superiority was observed in the rapid visual rehabilitation [84, 85]. The advantage of DMEK over DSAEK is its ability to achieve even faster and better visual outcomes, and this effect also applies to eyes with failed PKPs. At 6 months after surgery, the median BCVAs were 20/30, providing apparently better and more rapid effects than those achieved after DSAEK performed for failed PKPs in their recent report of 60 eyes, in which the median 6-month BCVA was 20/50 [85]. This finding is also in sharp contrast to the prolonged visual rehabilitation observed after repeat PKPs. Liarakos et al. reported a case of a patient who underwent DMEK after a failed primary PKP graft in the presence of a glaucoma drainage device (GDD) and concluded that DMEK could still be performed successfully in eyes with decompensated PKP grafts, even in the presence of a long GDD tube [87].

There are still limitations of DMEK compared with conventional PKP or even DSAEK, such as difficulty associated with the donor graft preparation and the achievement of complete graft attachment. Fortunately, recent technique improvements have reduced the occurrence of tissue loss and dramatically improved graft attachment rates. With the advantages of rapid visual recovery and fewer complications, this newer EK technique is still very attractive, especially for patients who are longing for rapid visual rehabilitation or at an increased risk for graft rejection. However, due to the relatively small sample sizes and heterogeneous nature of current research, further studies are warranted to verify these points and identify pre- and postoperative risk factors associated with outcomes.

### 4.3. Endothelial Keratoplasty versus Repeat Penetrating Keratoplasty after Failed Penetrating Keratoplasty

Recently, several observational studies have compared the outcomes of endothelial keratoplasty (EK) after a previously failed PKP with repeat PKP, although inconsistent results have been reported. Keane et al. collected data on 400 eyes with a second graft after a failed PKP that was performed initially for keratoconus or pseudophakic bullous keratopathy (PBK). The authors suggested that repeat PKP might deliver a better outcome than EK after failed PKP in terms of graft survival, but visual outcomes appeared to be equivalent across groups for the surviving graft [88]. In contrast, Ang et al. reported prospective results in 113 eyes with an initial indication of PBK. Graft survival was better for the EKs compared with the repeat PKPs up to 5 years of follow-up [89]. A retrospective study by Kitzmann et al. compared the data from PKP-PKP grafts (17 eyes) with those of PKP-EK (7 eyes) and found no significant difference in graft survival or visual acuity; however, there was a trend towards better postoperative visual acuity, a lower postoperative complication rate, and a higher graft survival rate in eyes that underwent DSAEK rather than repeat PKP for endothelial failure [90].

Thus, a meta-analysis was performed to compare graft survival, graft rejection, and the visual outcome of EK with repeat PKP after failed PKP, which included above-mentioned studies [91]. However, no significant differences in graft survival rate or visual outcome were observed between the two groups. As the data were obtained from multiple centers with different study population, varying surgical techniques, and surgeon experience levels, significant heterogeneity was present among the included studies, which might affect the results. However, the authors found that EK led to a significantly lower risk of graft rejection compared with repeat PKP after failed PKP, which was consistent with evidence from studies indicating a lower risk of endothelial rejection in primary EK than primary PKP.

Although these results were limited and inconclusive due to their small sizes and heterogeneity, the findings indicated that EK might be a better alternative to repeat PKP for second corneal transplantation with a lower graft rejection rate, especially for patients with prior graft failure resulting from endothelial edema or rejection. Further comparative studies with a larger size, longer follow-up, and well-described visual acuity outcome measurements are needed to improve our understanding of the benefits of EK versus repeat PKP for the treatment of failed PKP.

## 5. Conclusions

Any direct or indirect damage to the endothelium could cause the loss, morphological changes, and dysfunction of endothelial cells. Graft size and recipient factors such as indications, glaucoma, and glaucoma surgery are all highly associated with the occurrence of endothelial decompensation, while others are still under investigation. The pathogenesis can be concluded to be an acute and chronic loss of endothelium, and cell exchange between donor and recipient is at the core of chronic cell loss. DSAEK and DMEK are novel therapeutic strategies for failed PKP grafts and have potential superiorities compared with repeat PKP.

## Figures and Tables

**Table 1:** Studies on 10-year graft survival of PKP.

Study	Sample size	10-year survival rate (%)	Indication (*N*)	Follow-up time
Lass et al.^*∗*^ [3]	1090	74.5	Fuchs dystrophy (676)	10 years
			Pseudophakic or aphakic	
			Corneal edema (PACE) (369)	

Williams et al. [4]	16291	89	Keratoconus (6249)	<1 year (24%)
		37	Bullous keratopathy (4338)	1–6 years (58%)
		36	Failed previous graft (4227)	6–12 years (14%)
		66	Corneal dystrophy (2156)	12–18 years (4%)
				>18 years (1%)

Kelly et al. [5]	4834	89	Keratoconus (4834)	23 years

Anshu et al. [6]	901	90.7	Keratoconus (87)	36.8 ± 35.5 months.
		20.0	Fuchs' endothelial dystrophy (64)	
		33.5	Pseudophakic/aphakic bullous keratopathy (211)	
		—	Regraft (112)	

Sugar et al.^*∗*^ [7]	1090	80	Fuchs dystrophy (676)	12 years
		63	Pseudophakic or aphakic corneal edema (PACE) (369)	

Patel et al. [8]	388	80	Fuchs dystrophy (108)	20 years
			Keratoconus (83)	
			Pseudophakic corneal edema (73)	
			Aphakic corneal edema (68)	

Borderie et al. [9]	1144	64.4	Keratoconus (258)	40.5 ± 32.1 months
			Bullous keratopathy (449)	
			Endothelial dystrophies (121)	
			Regraft (87)	

Williams et al. [10]	10952	62	Keratoconus	Up to 15 years (mean follow-up time is not available)
			Regraft	
			Dystrophy	
			Aphakic bullous keratopathy	
			Pseudophakic bullous keratopathy (ratios are not available)	

Inoue et al. [11]	271	79.3	Keratoconus (64)	46.5 ± 35.1 months
			Bullous keratopathy (55)	
			Nonherpetic keratitis (49)	
			Graft failure (39)	
			Herpes keratitis (38)	
			Corneal dystrophies and degenerations (20)	
			Chemical burns (6)	

Thompson et al. [12]	3992	82	Primary grafts (3640)	43 ± 34 months
		92	Keratoconus (449)	
		90	Fuchs' dystrophy (908)	
		41	Regraft (352)	

Inoue et al. [13]	396	72.2	Overall (396)	46.6 ± 30.4 months
		98.8	Keratoconus (82)	
		76.9	Corneal dystrophy (26)	
		61.8	Regraft (68)	
		51.1	Bullous keratopathy (94)	

^*∗*^Data from the same group and same research population (Cornea Donor Study).

**Table 2:** Studies on 5-year and 10-year ECD after PKP.

Study	Sample size (the number of eyes)	Baseline mean ECD (95% confidence interval)	ECD (95% confidence interval)		ECD loss (%)	Precision of measurements	Follow-up time
			5 years	10 years			
Lass et al. [3]	1090	2661 (95% confidence interval not available)	—	614 (95% confidence interval not available)	77% from baseline to 5 years	Using a specular microscope	10 years
Kettesy et al. [23]	68	—	1501 (1442, 1560) (at examination time)		15.8% per year in first 2 years	Using a contact specular microscope	67.8 ± 74.1 months (range: 12–276 months)
Abbott et al. [26]	100	—	684 (at examination time)		—	With specular microscopy	Average follow-up of 17.4 years (range 10–36 years)
Linn et al. [27]	55	—	941(832, 1050) (at examination time)		50 % per 10 years	Using Zeiss noncontact endothelial lens	2.5 years to 35 years
						Minimum 41 of cells were counted	
Lass et al. [28]	567	2698 (95% confidence interval not available)	778 (95% confidence interval not available)	—	70% from baseline to 5 years	Using a specular microscope	5 years
Patel et al.^*∗*^ [8]	393	2973 (2919, 3027)	1191 (1116, 1266)	960 (876, 1044)	67 ± 17 (%) from baseline to 10 years	Using a specular microscope	20 years
Bourne et al.^*∗*^ [29]							
Patel et al.^*∗*^ [15]						The outlines or apices of at least 50 endothelial cells were digitized	
Ing et al.^*∗*^ [24]							
Borderie et al. [9]	1144	2264 (2247, 2281)	1058 (95% confidence interval not available)	865 (95% confidence interval not available)	41 ± 24% over the first 2 years, followed by a 10 ± 6% yearly decrease afterward	Using wide-field specular microscopy	40.5 ± 32.1 months
Inoue et al. [30]	15	—	—	998 (824, 1172)	12.1 ± 16.3% from 10 years to 20 years	Using nonapplanation contact specular microscopy	24.8 ± 3.1 years
						A minimum of 50 cells was counted in each photograph	
Lass et al. [31]	176	2695 (2498, 2890)	786 (616, 1343)	611 (502, 769)	76%	Using specular or confocal microscope	10 years
					95% confidence interval (70%, 82%)	With masked image quality grading and ECD determination	
					From baseline to 10 years	Minimum 15 of cells of each variable frame	

^*∗*^Data from the same group and same research population.

**Table 3:** Studies on risk factors for endothelial decompensation.

Study	Risk factors discussed in the researches		Sample size	Outcome
	Significant factors	Not significant factors		
Lass et al.^*∗*^ [3]	—	Donor status (history of diabetes)	1090	Graft failure and ECD at 10 years
Sugar et al.^*∗*^ [7]	Recipient factors (age, indication, glaucoma, smoking)	Recipient factors (race, sex, diabetes)	1090	Graft failure at 10 years
Williams et al. [10]	Donor status (age)	Donor status (cause of death)	10952	Graft failure
	Operative factors (graft size)	Operative factors (the impact of the surgeon)		
	Recipient factors (indication, glaucoma, number of previous ipsilateral grafts)	Recipient factors (age)		
Inoue et al. [11]	Operative factors (operation time)	Donor status(age)	271	Graft failure
	Recipient factors (age, lens status, transplant number)	Operative factors (graft size, suture technique)		
Xiao and Xie [16]	Recipient factors (indication)	—	151	Endothelial decompensation
Lass et al. [28]	Donor status(age, sex)	Donor status (race, cause of death, history of diabetes)	567	Endothelial cell loss at 5 years
	Operative factors (graft size)	Operative factors (method of retrieval, death to preservation time)		
		Recipient factors (age, race, sex, indication, glaucoma)		
Lass et al. [31]	Donor status (age, preoperative donor ECD)	Recipient factors (age)	176	ECD at 10 years
	Operative factors (graft size)			
Bertelmann et al. [37]	Recipient factors (glaucoma)	Donor status (age, preoperative donor ECD)	293	Endothelial cell loss at 36 months
		Recipient factors (indication)		
Wakefield et al. [38]	—	Donor status (age, preoperative donor ECD)	9415	Endothelial failure at 5 years
Yu et al. [39]	Donor status (age, preoperative donor ECD)	Donor status (sex, race, cause of death, history of diabetes)	377	Graft failure
	Recipient factors (age, indication, glaucoma)	Operative factors (death to preservation time, storage time)		
		Recipient factors (race, sex, laterality)		
Armitage et al. [40]	Donor status (sex)	Donor status (age, preoperative donor ECD)	3014	Graft failure at 5 years
	Operative factors (postoperative surgical procedures, donor/recipient trephine difference)	Operative factors (graft size, death to preservation time, storage time, suturing method)		
	Recipient factors (indication, glaucoma, infection)	Recipient factors (age, donor-recipient age difference, sex)		
Sugar et al.^*∗*^ [41]	Recipient factors (indication, glaucoma)	Operative factors (graft size)	1090	Graft failure at 5 years
		Recipient factors (age, sex, diabetes, smoking)		
Sugar et al.^*∗*^ [42]	—	Donor status (sex, cause of death, history of diabetes, preoperative donor ECD)	1090	Graft failure at 5 years
		Operative factors (method of retrieval, death to preservation time, endothelial cell damage		
		Descemet folds, epithelial defects)		
Gal et al.^*∗*^ [43]	—	Donor status (age)	1090	Graft failure at 5 years
Borderie et al. [44]	Donor status (age, preoperative donor ECD)	—	231	Graft failure at 1 year and ECD at 2 years
	Operative factors (graft size, storage time)			
	Recipient factors (age, indication, glaucoma)			
Chung et al. [45]	Operative factors (graft size)	—	90	ECD at 2 years
	Recipient factors (indication)			
Szentmary et al. [46]	Operative factors (the impact of the surgeon)		370	ECD

^*∗*^Data from the same group and same research population.
